# Outcome Predictors in Treatment of Yaws

**DOI:** 10.3201/eid1706.101575

**Published:** 2011-06

**Authors:** Oriol Mitjà, Russell Hays, Anthony Ipai, David Gubaila, Francis Lelngei, Martin Kirara, Raymond Paru, Quique Bassat

**Affiliations:** Author affiliations: Lihir Medical Centre, Lihir Island, Papua New Guinea (O. Mitjà, R. Hays, A. Ipai, D. Gubaila, F. Lelngei, M. Kirara, R. Paru);; Barcelona Centre for International Health Research, Barcelona, Spain (Q. Bassat)

**Keywords:** Yaws, Treponema pallidum subsp. pertenue, bacteria, skin lesions, serology, treatment failure, New Guinea, dispatch

## Abstract

To estimate failure rates after treatment with benzathine penicillin and to identify determinants of failure that affected outcomes for yaws, we conducted a cohort study of 138 patients; treatment failed in 24 (17.4%). Having low initial titers on Venereal Disease Research Laboratory test and living in a village where yaws baseline incidence was high were associated with increased likelihood of treatment failure.

Yaws is a tropical infection of the skin and bones caused by *Treponema pallidum* subsp*. pertenue* and is transmitted by direct, nonsexual contact with infectious lesions (*1**,**2*). Although a multinational mass eradication campaign in the 1950s greatly reduced the incidence of this disease (*3**–**5*), a resurgence of yaws has occurred in west and Central Africa, Southeast Asia, and the Pacific Islands (*6**–**9*). The currently recommended drug therapy for yaws is penicillin G benzathine, administered intramuscularly as a single dose of 1.2 million units (*3**,**5*). *T. pallidum* is a primary human pathogen that has eluded in vitro cultivation (*10*). Hence, although penicillin treatment failure has been reported for yaws (*11*), to date penicillin resistance has not been proven. Most serologically defined treatment failures are thought to be caused by either reinfection after treatment or patient-to-patient variation in the rate of decline in nontreponemal test titers after treatment (i.e., >4-fold decrease), rather than by relapse (*10*). The aim of this study was to estimate failure rates after treatment with benzathine penicillin and to identify determinants of failure that affected the outcome.

## The Study

We conducted a retrospective cohort study involving patients diagnosed with yaws at Lihir Medical Centre from January through September 2009. Ethics approval was obtained from the Papua New Guinea Ministry of Health Medical Research Advisory Committee. Diagnosis of yaws was based on correlation of the clinical findings, positive serologic results, and epidemiologic history. Patients <15 years of age whose mothers had negative treponemal test results at antenatal screening, with clinical evidence of early yaws (primary or secondary stage), and whose Venereal Disease Research Laboratory (VDRL) and *T. pallidum* hemagglutination test results were positive, were eligible to participate in the study. We included only case-patients with clear documentation of the village of residence, contact history, yaws clinical stage, clinical outcome, pretreatment titer, and at least 1 follow-up titer 12–15 months after treatment. We also estimated the minimum incidence rate for each of the 27 villages served by our hospital; a high incidence rate was defined as >1.5%. This percentage was calculated by dividing the number of new cases diagnosed at Lihir Medical Centre within the study period by the estimated population from each village, according to the local annual census. Treatment outcome was measured at a follow-up visit 12–15 months after treatment. Treatment failure was defined as the lack of a 4-fold decrease in VDRL titers at least 365 days after treatment.

**Figure 1:**
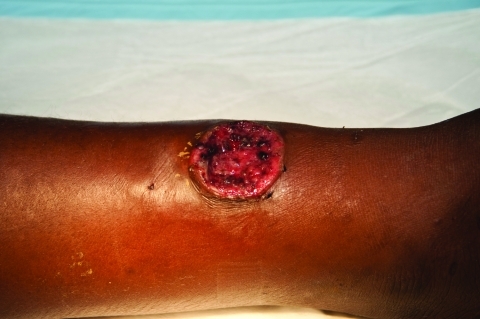
Painless ulcer with raised edges corresponding to a primary yaws skin lesion on an infant case-patient’s leg, Papua New Guinea, 2009. Source of photograph: Lihir Medical Centre, Dr Oriol Mitjá.

**Figure 2:**
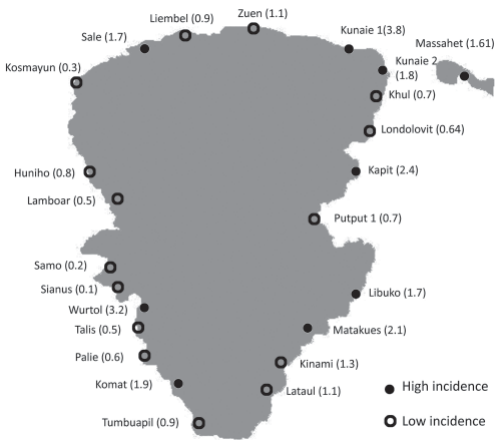
Map of Lihir Island, Papua New Guinea, showing incidence of infection in the 24 villages where cases of yaws were diagnosed, 2009. Lihir Medical Center is located in Londolovit village. Incidence proportions are shown within parentheses.

A total of 138 patients were identified during enrollment. Table 1 summarizes patient demographic characteristics, clinical signs and symptoms, laboratory results, and outcomes. Eighty-one (58.7%) persons displayed active primary cutaneous yaws lesions (Figure 1), and 63 (45.7%) exhibited signs of secondary stage yaws (hyperkeratotic skin papules or bone involvement). All patients were administered 3 doses of intramuscular benzathine penicillin 1×/wk, and only 6 (4.4%) children required 1 or 2 additional doses before initial symptoms disappeared. According to the estimated minimum incidence, in 9 villages the disease was classified as highly endemic, and in 15 villages, the disease was considered of low endemicity (Figure 2). Of the 138 analyzed case-patients, 90 (65.2%) persons came from a high minimum incidence village (HMIV) and 48 (34.8%) came from a low minimum incidence village (LMIV). Secondary stage lesions were found in 47 (52.2%) of 90 case-patients among the HMIV group and only in 16 (33.3%) of 40 case-patients in the LMIV group (p = 0.035). VDRL titers were significantly lower in case-patients in the HMIV group than in those in the LMIV group; 70% of case-patients in the HMIV group had a titer <32 (p = 0.026), as did 50% of those in the LMIV group. A positive association between a low initial VDRL titer and secondary stage disease was also found (79% of case-patients with secondary yaws had a low initial titer, compared with 51% who had primary yaws [p<0.01]). Overall, 24 (17.4%) case-patients experienced serologically defined treatment failure during follow-up, including 21 (23.3%) and 3 (6.3%) from the HMIV and LMIV groups, respectively. Multivariate analysis (Table 2) showed that only residence in a high incidence village (odds ratio 3.75, 95% confidence interval 1.02–13.76) and an initial VDRL titer <32 (odds ratio 4.05, 95% confidence interval 1.06–15.38) proved to be independent predictors for treatment failure.

**Table 1:** Demographic data, clinical signs/symptoms, laboratory results, and outcome after treatment of yaws in 138 case-patients, Papua New Guinea, January–September, 2009*

Characteristic	Total no. (%) patients, N = 138
Mean age, y (SD)	9.6 (4.4)
Male sex	81 (58.7)
VDRL titer	
16	54 (39.1)
32	33 (23.9)
64	42 (30.4)
128	9 (6.5)
Primary skin lesion	81 (58.7)
Secondary stage	63 (45.7)†
Family history	36 (26.1)
Treatment with IM penicillin G benzathine	138 (100.0)
Clinical healing	132 (95.7)
Concurrent disease	7 (5.1)‡
Seroconversion	63 (45.7)
Serologically defined treatment failure	24 (17.4)

*VDRL, Venereal Disease Research Laboratory; IM, intramuscular. †Includes 7 cases of early yaws osteoperiostitis, among whom were 3 patients with dactylilitis. ‡Includes 3 cases of *Plasmodium falciparum* malaria, 1 case of *P. vivax* malaria, 1 case of acute diarrhea diagnosed at the 12-month follow-up visit, and 2 case-patients with a chronic underlying disease (1 case of congenital heart disease and 1 case of chronic asthma).

**Table 2:** Association between characteristics of case-patients and *Treponema pertenue* infection treatment failure, Papua New Guinea, January–September 2009*

Characteristic	No. (%) patients treated			Univariate analysis			Multivariate analysis	
	Success, n = 114	Failure, n = 24		OR (95% CI)	p value		OR (95% CI)	p value
Mean age, y (SD)	9.54 (4.69)	10.13 (2.96)		0.58 (−1.39 to 2.56)	0.56		NA	NA
Male sex	69 (60.5)	12 (50.0)		0.65 (0.27–1.58)	0.34		NA	NA
Lived in a high-incidence village	69 (60.5)	21 (87.5)		4.57 (1.29–16.20)	0.02		3.75 (1.02–13.76)	0.04
Secondary yaws	49 (43.0)	14 (58.3)		1.86 (0.76–4.53)	0.17		1.01 (0.37–2.75)	0.99
Clinical healing	108 (94.7)	24 (100.0)		NA	0.59		NA	NA
Positive family history	27 (23.7)	9 (37.5)		1.93 (0.76–4.91)	0.17		1.91 (0.70–5.28)	0.20
VDRL titer <32	67 (58.8)	21 (87.5)		4.91 (1.39–17.41)	0.01		4.05 (1.06–15.38)	0.04

*p<0.05 was considered significant. OR, odds ratio; CI, confidence interval; NA, not applicable; VDRL, Venereal Disease Research Laboratory test.

## Conclusions

Serologically defined treatment failures occurred in ≈17% of case-patients in our series. Treatment failure could have been influenced by the capacity of the infecting agent to develop resistance to the antimicrobial drug used, or the failure could have been caused by other factors related to the human host. Our findings show that in Lihir, the factors predicting treatment failure after 12 months of drug therapy were the following: residence in a village where incidence of infection was high and initial VDRL titer was low. False-positive VDRL reactions, classically associated with viral and autoimmune diseases (*12*), are unlikely to be the cause of failure in our series, because no chronic underlying disease or concurrent febrile illnesses were registered in 23 (96%) of the case-patients who did not achieve a cure. Moreover, the strict epidemiologic criteria required (obtained through patient history) for inclusion in the study aimed to reduce the likelihood of false-positive results for syphilis. A VDRL titer of <32 dilutions proved to be a robust predictor of failure.

In our experience, this low titer is also associated with longer lasting infections and is more commonly found in high incidence villages (as are longer lasting infections). Even after multivariate analysis, clarifying the role of these confounding factors is difficult. We suspect that the true factor at work here rests upon the assumption that a chronic infection is more difficult to resolve. The tissue-to-plasma ratios for bone penetration are usually between 0.1 and 0.3 for penicillins and are even lower for cortical bone than for cancellous bone (*13*). On the basis of these ratios, treponemes that invade the bone would encounter subtherapeutic levels of penicillin, which could simply lead to persistent infection or even provide selective pressure for mutations for penicillin resistance. On the other hand, the risk for reinfection caused by repeated contact with infected children seems to be a pivotal factor in predicting treatment failure. The high number of asymptomatic persons or persons with few symptoms in high prevalence areas is the main reservoir of the infection and a known obstacle to achieving the eradication of yaws. A limitation of our study is the use of incidence rates derived from hospital-based detected cases, which likely underestimated the real incidence of infection. Also, the calculation of this proportion might be less precise because we did not take into account factors such as the distance of the village from the health center or the proportion of children to adults in a particular village.

On the basis of our findings, we anticipate that a community-based strategy will be required to effectively control yaws on Lihir Island. The current strategy for eradication of yaws in areas where the disease is moderately endemic (prevalence <5%) is to treat patients with active cases and their contacts. In our experience, most children did not have a family history of yaws. Thus, the disease likely is not clustered in households but, rather, transmission is more likely to occur among children in the community, in schools, and in other public places. Future eradication programs will need to take into account all epidemiologic, biological, and pharmacologic factors, along with the practical considerations of a mass campain to deliver and administer drugs in isolated and underresourced communities. In this context, the potential treating yaws with oral, single-dose therapy, for example, with azithromycin, should be explored.
